# RNA Binding Protein Vigilin Collaborates with miRNAs To Regulate Gene Expression for *Caenorhabditis elegans* Larval Development

**DOI:** 10.1534/g3.117.043414

**Published:** 2017-06-02

**Authors:** Rebecca A. Zabinsky, Brett M. Weum, Mingxue Cui, Min Han

**Affiliations:** * Howard Hughes Medical Institute, University of Colorado at Boulder, Boulder, Colorado 80309; † Department of Molecular, Cellular, and Developmental Biology, University of Colorado at Boulder, Boulder, Colorado 80309

**Keywords:** miR-2, miR-45, miR-51, miR-83, miRNA, vgln-1, Vigilin

## Abstract

Extensive studies have suggested that most miRNA functions are executed through complex miRNA-target interaction networks, and such networks function semiredundantly with other regulatory systems to shape gene expression dynamics for proper physiological functions. We found that knocking down *vgln-1*, which encodes a conserved RNA-binding protein associated with diverse functions, causes severe larval arrest at the early L1 stage in animals with compromised miRISC functions (an *ain-2/GW182* mutant). Through an enhancer screen, we identified five specific miRNAs, and miRNA families, that act semiredundantly with VGLN-1 to regulate larval development. By RIP-Seq analysis, we identified mRNAs that are directly bound by VGLN-1, and highly enriched for miRNA binding sites, leading to a hypothesis that VGLN-1 may share common targets with miRNAs to regulate gene expression dynamics for development.

miRNA-mediated gene silencing is crucial for all aspects of cellular processes, including animal development, metabolism, stress responses, and neuronal behaviors (Ambros 2004; Bartel 2009). Misregulation of miRNAs has been linked to numerous human diseases (Li and Kowdley 2012; Mendell and Olson 2012; Orellana and Kasinski 2015). Although disrupting *lin-4* and *let-7* produces a robust developmental phenotype (Vella and Slack 2005), the vast majority of miRNAs and miRNA families are not essential for development or viability (Miska *et al.* 2007; Alvarez-Saavedra and Horvitz 2010). Due to redundancy and complex interactions between multiple miRNAs and multiple targets, single gene genetic methods are not sufficient to elucidate the physiological roles of many miRNAs. Our laboratory, and others, have provided evidence to support the idea that miRNAs function redundantly with other gene regulatory mechanisms to ensure developmental robustness (Hammell *et al.* 2009a; Brenner *et al.* 2010; Vidigal and Ventura 2014; Weaver *et al.* 2014; Ren *et al.* 2016).

Our previous study demonstrated that compromising overall miRISC function by mutating one of two *C. elegans* GW182 proteins dramatically enhanced developmental defects associated with RNAi knockdown of a large number of genes (Weaver *et al.* 2014). In many cases, the phenotypes were synergistic. One such gene identified, C08H9.2, encodes a putative RNA binding protein homologous to mammalian HDLBP or Vigilin (Weber *et al.* 1997; Rohwedel *et al.* 2003). We thus named the C08H9.2 gene *vgln-1* (ViGiLiN).

Studies in yeast, *Drosophila*, and humans have shown that Vigilin genes are involved in various cellular processes, including cell division, mRNA transport, RNA editing, translation, heterochromatin formation, and cancer (Weber *et al.* 1997; Frey *et al.* 2001; Li *et al.* 2003; Wang *et al.* 2005; Hogan *et al.* 2008; Zhou *et al.* 2008; Woo *et al.* 2011; Gelin-Licht *et al.* 2012; Mitchell *et al.* 2013; Molyneux *et al.* 2014; Otsuka *et al.* 2014; Yang *et al.* 2014; Mobin *et al.* 2016). The diversity of functions and phenotypes associated with this RNA binding protein suggests that the Vigilin proteins have diverse RNA targets.

The functional relationship between Vigilin and miRNAs has not been previously explored. However, RNA binding proteins have been shown to directly or indirectly influence miRNA regulation of target mRNAs for specific cellular and physiological functions (Hammell *et al.* 2009b; van Kouwenhove *et al.* 2011; Akay *et al.* 2013; Ciafrè and Galardi 2013). It is not clear whether a more general RNA-binding protein, or domain, is commonly involved in miRNA-mediated silencing. In this study, we applied genetic enhancer analysis to investigate the functional relationship between VGLN-1 and miRNAs during *Caenorhabditis elegans* larval development. Our study uncovered previously unknown roles of both VGLN-1 and several specific miRNAs in larval development, and the data suggest potential collaborative regulation by VGLN-1 and miRNAs on common mRNA targets.

## Materials and Methods

### RNA interference (RNAi)

RNAi constructs targeting either the 3′end or 3′UTR of *vgln-1* were constructed by amplifying 500 bp of the region of interest, cloning them into the L4440 vector, and transforming into HT115 bacteria. Primer sequences were:

3′ UTR forward (with *Eag*I), ataaCGGCCGGatgattcaaccaacgtcaataac.
3′UTR reverse (with *Xho*I), atgcCTCGAGTtttgattacaccaattcagtttatta.
5′end forward (with *Xba*I), ataaTCTAGAAGGACAACTCCAGCGAGGA.
5′end reverse (with *Hin*dIII), ataaAAGCTTCTCGACGTTGTCAAGCAAGA.

All other RNAi bacteria were obtained from the ORFeome RNAi feeding library (Reboul *et al.* 2003) and the *C. elegans* RNAi library (Kamath *et al.* 2003). All RNAi was delivered by feeding. An RNAi sensitive strain NL2099 with a *rrf-3* mutation was used (Supplemental Material, Figure S1A in File S1). To achieve a strong *vgln-1* (RNAi) effect, bacteria were picked from a fresh colony, and grown for ∼8 hr with 100–200 μg/ml ampicillin and 15 μg/ml tetracycline. Alternatively, an overnight culture was diluted and grown for 4 hr. After spotting on nematode growth medium (NGM) plates with 0.4 mM isopropyl-β-d-thiogalactoside (IPTG) and 100 μg/ml ampicillin, plates were allowed to dry for 4 d before adding L4 stage worms. After the worms reached gravid adult stage, they were transferred to new RNAi plates and allowed to lay eggs for 24 hr. The progeny were then scored ∼40 hr later for the L1 arrest phenotype.

For brood size analysis, one L4 stage nematode of the double mutant was randomly selected from each plate (NGM medium) and transferred to a fresh plate. A total of 18 worms was counted. The worms were transferred to fresh plates daily until they stopped laying eggs, and the number of eggs was recorded every day. The nematode brood size was determined based on the sum of total eggs laid by individual hermaphrodites.

### Generating transgenic strains

Plasmids were obtained from Addgene. pDD162 (Peft-3::Cas9 + Empty sgRNA) was a gift from B. Goldstein (Addgene plasmid # 47549) (Dickinson *et al.* 2013). pCFJ350—MCS(*ttTi5605*, II) and pMA122—*peel-1* negative selection were gifts from E. Jorgensen (Addgene plasmid # 34866 and # 34873) (Frøkjær-Jensen *et al.* 2012). A short guide RNA to target Cas9 to the endogenous *vgln-1* locus was designed and cloned into pDD162. A homologous recombination template to replace the *vgln-1* gene with *unc-119* from pCFJ350 was cloned into pBIISK(+). Selectable markers were used to isolate a null allele based on the published protocol (Dickinson *et al.* 2013). Loss of the *vgln-1* gene in strain MH5004 *vgln-1*(*kuIs104*) was further confirmed by PCR.

The transcriptional reporter *P_vgln-1_*::*gfp* (*kuIs103*) (strain MH4922) consists of the *vgln-1* promoter driving GFP with the *vgln-1* 3′UTR. The fusion reporter construct *P_vgln-1_*::*vgln-1*::*gfp* (*kuIs102*) (strain MH4920) consists of the *vgln-1* promoter driving the *vgln-1* cDNA fused to GFP with the *vgln-1* 3′UTR. These constructs were cloned into the pCFJ350 vector with a functional *unc-119* gene from *Caenorhabditis briggsae* and then introduced into the genome of *unc-119* mutant worms by microparticle bombardment (Merritt 2010). Three out of four lines obtained for each reporter exhibited high levels of GFP expression. These strains were also used for CLIP and RIP-Seq.

### Crosslinking and immunoprecipitation (CLIP)

Mixed-stage worms containing either *P_vgln-1_*::*vgln-1*::*gfp* (*kuIs102*) (strain MH4920) or *P_vgln-1_*::*gfp* (*kuIs103*) (strain MH4922) were grown on NGM plates with HB101 bacteria, collected by washing with M9 buffer, and irradiated by UV (Broughton and Pasquinelli 2013) to crosslink protein to RNA, and then stored at −70°. Worms were lysed by grinding in liquid nitrogen and immediately resuspended in lysis buffer (10 mM Tris-Cl pH 7.5, 150 mM NaCl, 0.5 mM EDTA, 0.5% NP-40, plus addition of complete mini Roche protease inhibitor pills, Thermo Fisher RNaseOut, 100 mM PMSF). Immunoprecipitation of protein crosslinked to RNA was performed with GFP-Trap A fusion proteins coupled to agarose beads according to the vendor’s protocol (Chromotek). RNA was digested, radiolabeled, and visualized as described (Moore *et al.* 2014).

### RNA immunoprecipitation (IP) and sequencing (RIP-Seq)

Worms containing either *P_vgln-1_*::*vgln-1*::*gfp* (*kuIs102*) (strain MH4920) or *P_vgln-1_*::*gfp* (*kuIs103*) (strain MH4922) were grown on large peptone-enriched plates spotted with HB101. Worms were collected using S-basal medium, bleached, and synchronized for 30 hr in S-basal without cholesterol. The synchronized L1s were then flash-frozen in liquid nitrogen. Worms were lysed by grinding in liquid nitrogen, and immediately resuspended in lysis buffer (10 mM Tris-Cl pH 7.5, 150 mM NaCl, 0.5 mM EDTA, 0.5% NP-40, plus addition of complete mini Roche protease inhibitor pills, Thermo Fisher RNaseOut, 100 mM PMSF). IP was performed with GFP-Trap A fusion proteins coupled to agarose beads according to the vendor’s protocol (Chromotek). Prior to IP, 100 μl of the lysate was removed and used for total RNA samples (Total). Following IP, RNA from strain MH4920 (IP) or strain MH4922 (CTRL) was digested using Trizol (Molecular Research Center) and 4-bromoanisole (Sigma). IP and CTRL immunoprecipitations were performed synchronously, and repeated for a total of three biological replicates. Library preparation of the resulting RNA samples was performed by the Biofrontiers Institute NGS Core Facility (University of Colorado at Boulder) using the Lexogen SENSE Total RNA-Seq Library Prep Kit. Sequencing was then performed on an Illumina HiSequation 2000 using a read size of 1 × 100.

Three libraries of VGLN-1::GFP (IP), two libraries of GFP (CTRL), and two libraries of total RNA (Total, pre-IP) were sequenced. Analysis was performed using Galaxy (Afgan *et al.* 2016). Quality controls were analyzed by FastQC from The Babraham Bioinformatics group at https://www.bioinformatics.babraham.ac.uk/projects/fastqc/. The resulting raw reads were processed using the FASTX-Toolkit from the Hannon lab at http://hannonlab.cshl.edu/fastx_toolkit/download.html. First reads were trimmed by removing the first 9 bp (as recommended by Lexogen), followed by removing reads with an overall quality < 30. Reads were then mapped to the genome using TopHat (Trapnell *et al.* 2009) and the Illumina iGenome WBCel235.

Differential analysis was performed in multiple ways. Mapped reads were run through both the CuffLinks pipeline with CuffDiff (Trapnell *et al.* 2013) using standard settings, and in parallel through DESeq2 (Love *et al.* 2014) using HTSeq (Anders *et al.* 2015) for read counts. In both cases, comparisons were made between replicates of VGLN-1::GFP (IP) *vs.* GFP (CTRL); fold enrichment represents the ratio of (IP) over (CTRL). Additionally, IP enrichment analysis was performed using HTSeq and DESeq2 according to the following formula:

$$\frac{AdjustedCounts\left(IP\right)-AdjustedCounts(CTRL)}{AdjustedCounts(Total)}$$

where “adjusted counts” for any transcript refers to:

$$\frac{Raw\;Number\;of\;Mapped\;Reads\;for\;Specific\;Transcript}{Total\;Number\;of\;Reads\;in\;Library}$$

Resulting CuffDiff results, DESeq2 results, and IP enrichment results were then compared for enrichment.

### 3′ UTR analysis

Resulting enriched targets were then analyzed for number of conserved miRNA seed sites per 1 kb of 3′ UTR length. 3′ UTR information, conserved miRNA site predictions (≥7mer), and 3′ UTR length information was all obtained from TargetScanWorm (Jan *et al.* 2011). For genes containing >1 3′ UTR isoform, the median number of conserved miRNA seed sites between all available isoforms was used. Conserved miRNA sites per 1000 bp were calculated by:

$$\left(\frac{median\;number\;miRNA\;sites\;}{median\;3^{’}UTR\;length}\;\right)*\text{1}000$$

The conserved sites per 1000 bp of enriched targets identified by RIP-Seq were then filtered based on annotated expression during the L1 stage of development, and compared to the conserved sites per 1000 bp of all available 3′ UTRs in genes expressed during the L1 stage of development (Spencer *et al.* 2011).

### Data availability

All strains used are listed in Table S1. Unless otherwise stated, strains were maintained and manipulated, as previously described (Brenner 1974; Stiernagle 2006), on NGM agar with *Escherichia coli* OP50 as a food source at 20°. Data from miRNA enhance screen, RIP-Seq and DESeq analyses, miRNA seed site analysis of VGLN-1 bound mRNAs, and analysis of VGLN-1-IP enrichment relative to total RNA are shown in Table S2, Table S3, Table S4, and Table S5, respectively.

## Results

### VGLN-1 is essential for development when miRISC function is compromised

While worms containing a loss of function (*lf*) *ain-2* mutation, or treated with *vgln-1*(*RNAi*), are superficially wild type, treating *ain-2*(*lf*) with *vgln-1*(*RNAi*) led to synthetic developmental arrest of F1 progeny at the first larval stage (Figure S1A in File S1) (Weaver *et al.* 2014). To eliminate potential off-target effects, we performed RNAi analysis using three constructs targeting two nonoverlapping coding regions, and the 3′UTR region (*Materials and Methods*). RNAi treatment using each construct caused a partial L1 arrest phenotype in an RNAi-sensitive background, and the phenotype was dramatically enhanced by *ain-2*(*lf*), supporting the view that the phenotype is due to silencing of *vgln-1* expression (Figure S1A in File S1).

**Figure 1:**
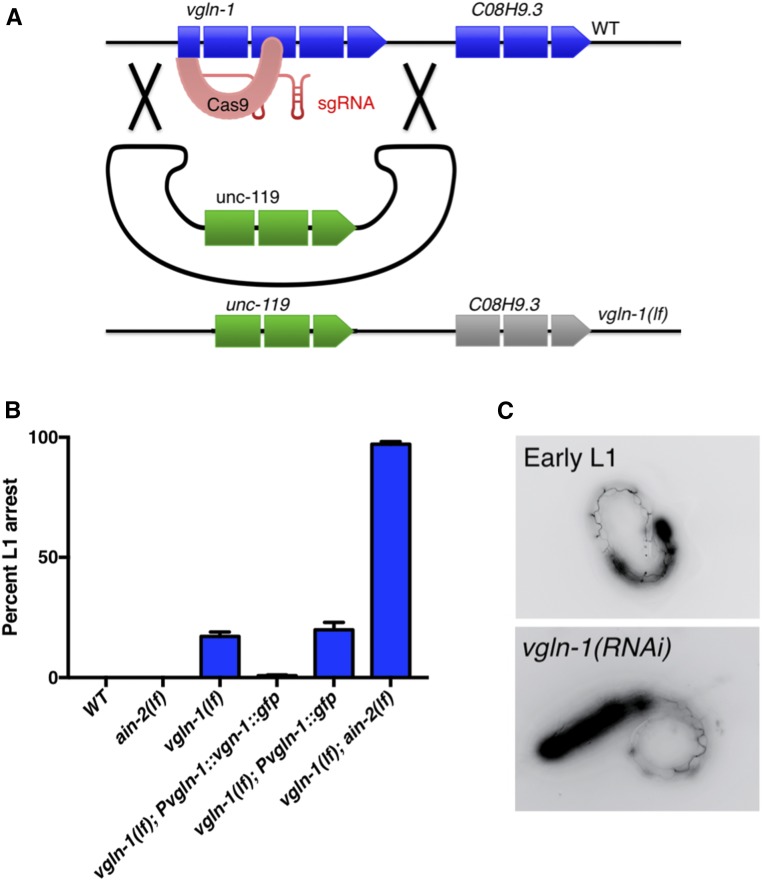
VGLN-1 is essential for postembryonic development when miRISC is compromised. (A) Cartoon illustration of the construct design of *vgln-l*(*lf*) using CRISPR/Cas9. (B) Percentage of hatched animals arrested at the early L1 stage of indicated genotype. Loss of *ain-2* dramatically enhances the phenotype of *vgln-1*(*lf*). The *vgln-1*::*gfp* fusion gene driven by the *vgln-1* promoter is functional. Error bars represent SE of the proportion. Number of animals examined = *N*. *vgln-1*(*lf*) *N* = 410, *ain-2*(*lf*) *N* = 323, *vgln-1*(*lf*);*ain-2*(*lf*) *N* = 251, *vgln-1*(*lf*);*Pvgln-1*::*vgln-1*::*gfp* *N* = 824, and *vgln-1*(*lf*);*Pvgln-1*::*gfp* *N* = 167. (C) Images of arrested L1 larvae expressing an AJM-1::GFP fusion protein that marks adherens junctions between seam cells. The pattern of seam cells (Knight *et al.* 2002) indicates that *rrf-3*;*vgln-1*(*RNAi*) animals arrest development within 3 hr of hatching.

To facilitate further genetic analysis, we generated a *vgln-1* null allele by applying the CRISPR gene knockout method (Figure 1A) (Dickinson *et al.* 2013). This *vgln-1*(*kuIs104*, *lf*) single mutant exhibited 20% L1 arrest, and a slight developmental delay. However, in combination with *ain-2*(*lf*), the double mutant exhibited a severe but incomplete sterile phenotype (although average brood size was >100, <75% of eggs hatched after 72 hr) (Figure S1, B and C in File S1). Among the progeny, 97% exhibited L1 arrest (Figure 1B) consistent with *vgln-1*(*RNAi*) tests. The partial L1 arrest phenotype of the *vgln-1*(*lf*) mutant was suppressed by expression of an integrated, translational fusion reporter *P_vgln-1_*::*vgln-1*::*gfp* (*kuIs102*) (Figure 1B). These results indicate that the putative RNA binding protein VGLN-1 functions in early larval development and reproductive development, and is essential when miRISC function is compromised.

To better characterize the developmental arrest that occurs with knockdown of both *vgln-1* and *ain-2*, we observed the pattern of an AJM-1::GFP reporter (Knight *et al.* 2002). During postembryonic development, seam cells divide in a characteristic pattern, and this reporter marks adherens junctions between seam cells. The pattern observed in arrested *vgln-1*(*RNAi*) larvae corresponds to 3 hr after hatching (Figure 1C), visually similar to that observed in larvae under starvation-induced diapause (Baugh and Sternberg 2006). We also found that intestinal tubulogenesis and apical membrane polarity (indicated by ERM-1::GFP), that have been found to be disrupted in some L1 arrested mutants (Zhang *et al.* 2012; Zhu *et al.* 2015), were unaffected by loss of *vgln-1* (Figure S2 in File S1).

### VGLN-1 binds to RNA and is broadly expressed throughout C. elegans development

Based on homology, and the presence of 14 KH domains, VGLN-1 is predicted to function as an RNA binding protein. To test its RNA binding property, proteins and RNAs were identified via UV CLIP from GFP tagged VGLN-1 strains described below and in *Materials and Methods*. The radiolabeled RNA from IP was detected by electrophoresis, and the RNA-protein complex was found to be sensitive to RNase treatment (Figure S3 in File S1), confirming that VGLN-1 binds RNA.

We found that the translational fusion reporter construct *P_vgln-1_*::*vgln-1*::*gfp* (*kuIs102*) was expressed ubiquitously throughout development, notably in the intestine [consistent with previous observations (McGhee *et al.* 2007)], spermatheca, and vulva (Figure S4 in File S1). The reporter appears to be mostly excluded from the nucleus suggesting that VGLN-1 functions mainly in the cytoplasm.

### Specific miRNAs and miRNA families act with VGLN-1 to regulate early larval development

**Figure 2:**
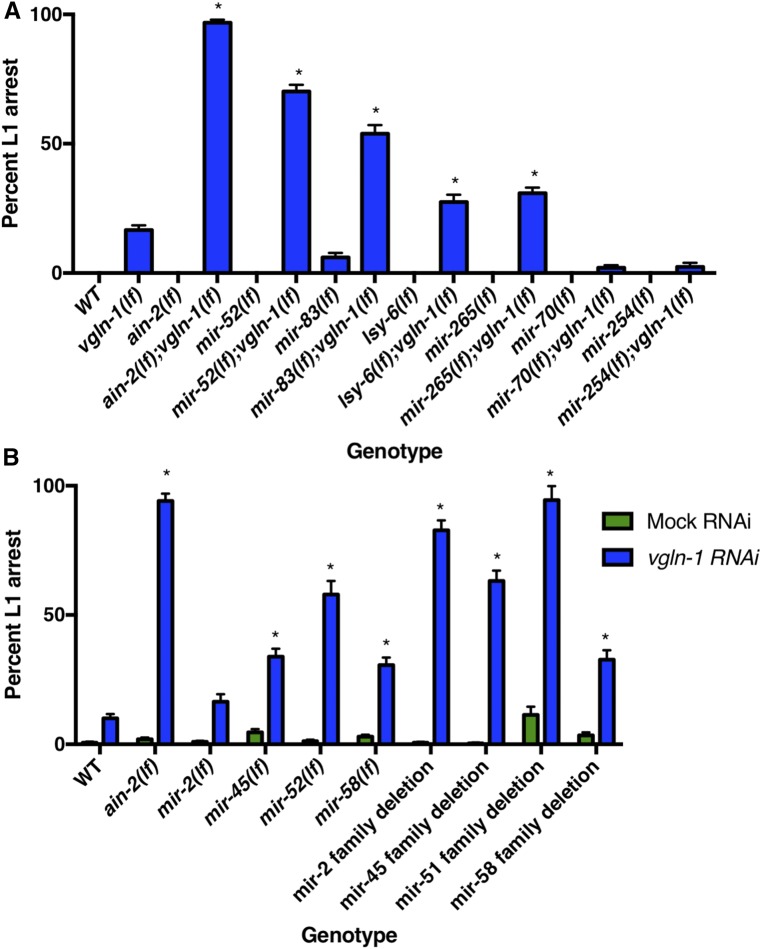
Specific miRNAs and miRNA families cooperate with Vigilin to regulate development. (A) Loss-of-function mutations in multiple miRNAs enhance the L1 arrest phenotype of *vgln-1*(*lf*). *mir-70* and *mir-254* were identified in our RNAi screen, but the double mutants did not exhibit a synthetic phenotype. Error bars represent SE of proportion. *N* = 85–777 worms counted. * *P* < 0.001 (Fisher’s exact tests comparing the proportion indicated to WT and to *vgln-1*(*lf*)). (B) While knockout of single miRNAs miR-2, miR-45, and miR-52 also enhanced the L1 arrest phenotype of *vgln-1*(*RNAi*), knockout of the entire miR-2 family, miR-45 family, mir-58 family, and a subset of the miR-51 family further enhanced the L1 arrest phenotype of *vgln-1*(*RNAi*). *P < 0.0001 [Fisher’s exact tests comparing the proportion indicated to WT on *vgln-1*(*RNAi*)].

Based on the synergistic effect between *vgln-1*(*lf*) and *ain-2*(*lf*) on larval lethality, we hypothesized that VGLN-1 collaborates with multiple miRNAs to regulate many mRNA targets to promote larval development. To identify these specific miRNAs, we screened all the available *C. elegans* miRNA deletion strains for prominent enhancement of the L1 arrest phenotype using *vgln-1*(*RNAi*). Our screen identified several miRNAs of which the deletion mutations significantly enhanced the L1 arrest phenotype of *vgln-1*(*RNAi*) (Table S2). To exclude the possibility that the enhancement is due to increased sensitivity to RNAi in these miRNA deletion strains (Massirer *et al.* 2012), we constructed *vgln-1*(*lf*);*miRNA*(*lf*) double mutants, and confirmed that deletion of *mir-52*, *mir-83*, *lsy-6*, and *mir-265* prominently enhanced the L1 arrest phenotype of *vgln-1*(*lf*) (Figure 2A).

miR-52 is part of the miR-51 family in *C. elegans*, which shares the same seed sequence with the highly conserved miR-99/100 family (Grimson *et al.* 2008). The miR-51 family is one of the few miRNA families that displays a severe developmental defect (embryonic lethality) when all members are knocked out (Alvarez-Saavedra and Horvitz 2010; Shaw *et al.* 2010; Brenner *et al.* 2012). miR-52 is known to be highly expressed at the L1 stage as well as L4, and is expressed significantly higher than other miR-51 family members (Lau *et al.* 2001; Lee and Ambros 2001; Martinez *et al.* 2015). The expression difference might explain why loss of miR-52, but not closely related family members, enhances the *vgln-1*(*lf*) phenotype (Table S2). miR-83 is expressed throughout larval development in several tissues, including neurons and the intestine, and shares a seed sequence with miR-49 (Martinez *et al.* 2015). LSY-6 is well characterized for its role in chemosensory receptor expression and neuronal asymmetry, and its expression was detected in <10 neurons (Johnston and Hobert 2003). miR-265 is also expressed in a few head neurons (Martinez *et al.* 2015). Although seed sequence alone predicted many potential mRNA targets for LSY-6 and miR-265, the specificity of their functional relationship with these mRNAs remains unknown (Didiano and Hobert 2006, 2008; Garcia *et al.* 2011). Baring the caveats of potential background mutation effect in the miRNA deletion strains (see *Discussion* below), our genetic analysis likely uncovered previously unknown functions associated with miR-52, miR-83, LSY-6, and miR-265 in early larval development.

To identify interactions between VGLN-1 and miRNAs of multi-member miRNA families, we tested whether knockdown of entire miRNA families could enhance the *vgln-1*(*RNAi*) phenotype. We found that miR-2, miR-45, miR-51, and miR-58 family deletions all resulted in significant larval arrest phenotypes on *vgln-1*(*RNAi*) (Figure 2B). Both miR-2 and miR-45 family deletions have a slight egg retention phenotype, but no developmental phenotypes (Alvarez-Saavedra and Horvitz 2010). While most of these miRNA families exhibited a more severe phenotype relative to the single miRNA deletions, the miR-58 family phenotype was very similar to that of the single mutant (Figure 2B), so our further analysis focused on the single miRNA miR-58. Therefore, our analysis has also identified novel roles for the miR-2, miR-45, miR-51, and miR-58 families of miRNAs in early larval development. Based on these comprehensive genetic results, we focused further analysis on the miR-2, miR-45, and miR-51 families, as well as miR-58 and miR-83.

We need to point out that it is possible that the synergistic lethality in strains combining a mutation of specific miRNA and *vgln-1*(*lf*) [or *vgln-1*(*RNAi*)] was due to a background mutation in the miRNA deletion strain created by chemical mutagenesis. The fact that *lsy-6* is known to be expressed only in a few neurons invites a more serious consideration of this caveat. However, the possibility that the effects of all or most of these miRNA mutants being the effect of second site mutations is highly unlikely for several circumstantial reasons. First, the enhancement of larval lethality is consistent with the enhancer phenotype associated either *ain-2*(*lf*) or *ain-1*(*lf*) mutation, both of which are known to specifically compromise overall miRISCs functions (Ding *et al.* 2005; Zhang *et al.* 2007; Weaver *et al.* 2014). Second, in the case of miR-58, similar phenotypes are associated with single miRNA mutant and deletion of corresponding miRNA family. Finally, as shown below the binding sites of the five miRNA families are enriched in VGLN-1 binding RNA at a particularly high level.

### VGLN-1 interacts with a set of mRNAs that are enriched for miRNA binding sites

**Figure 3:**
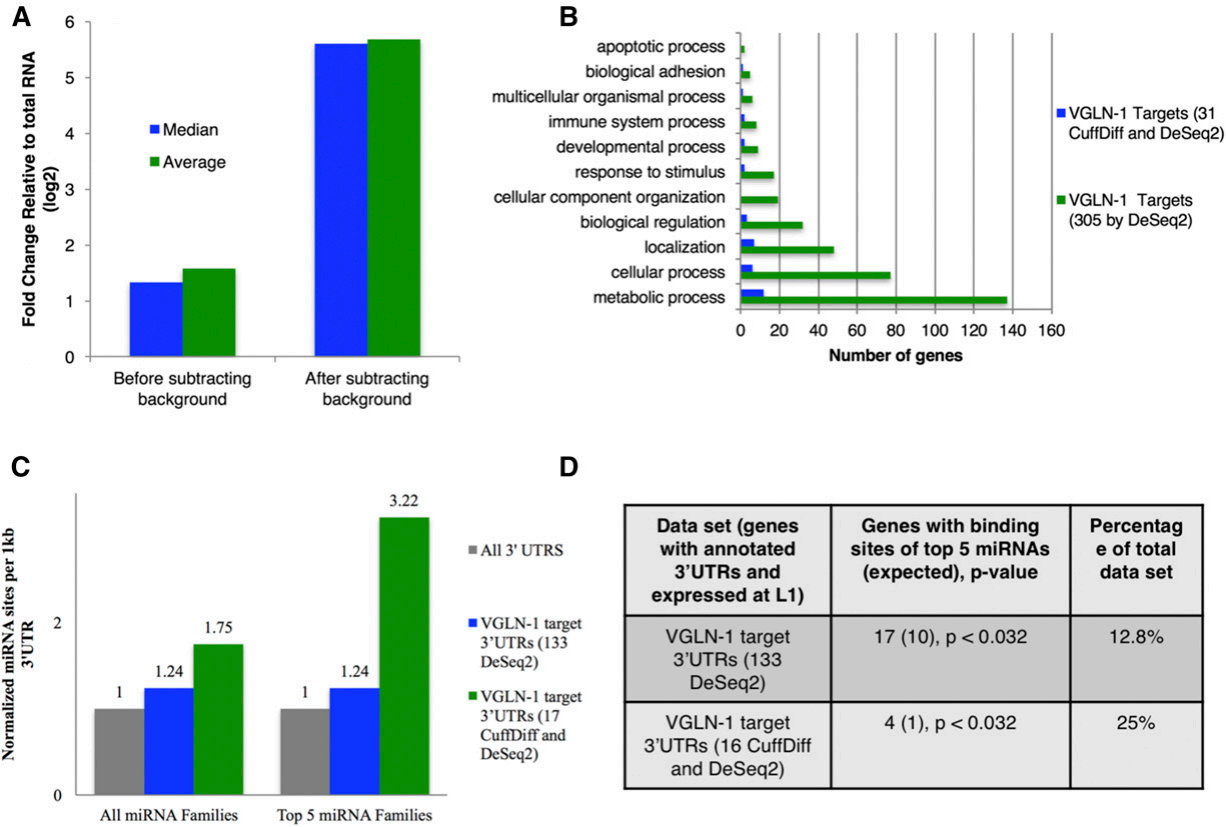
Identification of VGLN-1 bound mRNAs and enrichment for miRNA binding sites in these mRNA 3′UTRs. (A) Removing background identified by our GFP control improved identification of VGLN-1 bound RNAs that are enriched in the IP relative to total RNA. Median and average fold change of VGLN-1 bound RNAs from RIP-Seq was highly elevated relative to the median and average fold change of all other mRNAs when accounting for background measured by a control GFP IP. (B) Functional GOTerm analysis of VGLN-1 target mRNAs revealed an enrichment for genes involved in metabolic processes. (C) VGLN-1 bound mRNAs have a high density of all miRNA binding sites, and of miRNAs that we show genetically interact with *vgln-1* (miR-2, miR-45, miR-51, miR-83, and miR-58 families). (D) Enrichment and percent of gene sets with miRNA sites of the top five families that we show to interact genetically with *vgln-1*. *P*-values were calculated by a hypergeometric distribution. http://nemates.org/MA/progs/overlap_stats.html.

To identify VGLN-1-bound mRNAs, we employed RNA IP and high-throughput sequencing (RIP-Seq) (Zhao *et al.* 2010), using an anti-GFP antibody on samples from strains expressing either VGLN-1::GFP or control GFP. The resulting reads from three biological replicates were trimmed and mapped to the genome. We then performed differential expression analysis using both the Cufflinks suite and the DESeq2 R software suite to compare the FPKM values or reads of the VGLN-1::GFP IP to that of the GFP control IP. Together, these approaches yielded 304 enriched targets (*q* < 0.1, Table S3). Targets identified by DESeq2 encompassed the 31 targets identified by CuffDiff. Relative to the median and average fold change of all mRNAs, the median and average fold change of targets in the VGLN-1 IP was highly elevated when accounting for background through use of the GFP IP controls (Figure 3A and Table S5). GOTerm analysis of the 304 targets identified revealed enrichment in genes associated with metabolic processes, localization, and development (Figure 3B). The striking enrichment of genes involved in metabolic processes, which is consistent with the reported roles of human Vigilin (Mobin *et al.* 2016), may suggest a potential connection between metabolic abnormality and larval arrest.

3′UTR analysis of our 133 targets, and 17 high confidence targets known to be expressed at L1 (Spencer *et al.* 2011), revealed a large increase in the density of conserved (7mer or 8mer) miRNA seed sites relative to annotated UTRs of mRNAs expressed at the L1 stage of larval development. We observed an increase in total seed sites (1.2-fold), and a more dramatic increase in seed sites for the miRNA families that we show interact genetically with *vgln-1* (3.2-fold) (Figure 3C). The enrichment of predicted targets of these miRNA families in our target gene sets was determined to be significant by a hypergeometric distribution test (Figure 3D). The five families tested for enrichment in Figure 3, C and D were miR-2, miR-45, miR-51, miR-83, and miR-58 families. *mir-265* and *lsy-6* were excluded from these analyses due to their limited spatial expression, and extensive list of predicted mRNA targets (Johnston and Hobert 2003; Martinez *et al.* 2015). These results indicate that many of the 3′UTRs of VGLN-1 targets are likely recognized by miRNAs, and, more specifically, miRNAs that we show genetically interact with *vgln-1*. Table S4 details the specific seed sites found within each of these target mRNA 3′UTRs.

## Discussion

Our interrogation of genetic redundancy has revealed key players in early larval development. Genetic analysis has revealed overlapping, previously unknown roles for VGLN-1 and several individual miRNAs, and miRNA families, during early larval development. Based on the identification of VGLN-1-bound mRNAs and miRNA target predictions, both VGLN-1 and these miRNAs are expected to act on a large number of RNA targets. The diversity of functions associated with these VGLN-1-bound mRNAs implied that VGLN-1 acts in multiple cellular events (Figure 3B). For example, the association of many of these mRNAs with metabolism is consistent with the observation that human Vigilin regulates the expression of many metabolic genes (Graham and Oram 1987; Mobin *et al.* 2016).

Alone, *C. elegans vgln-1* was also reported as one of 25 genes identified in a enhancer screen for a potential role in developmental timing (Hayes and Ruvkun 2006). Phenotypes from genome-wide RNAi analysis also suggested potential functions of *vgln-1* in body morphogenesis, locomotion, and larval development (Kamath *et al.* 2003; Simmer *et al.* 2003; Ceron *et al.* 2007; Cantacessi *et al.* 2009). The sensitized genetic background of *ain-2*(*lf*) used in our study, where miRNA function is compromised, critically facilitated the exposure of the role of VGLN-1 in *C. elegans* larval development. Likewise, the sensitized genetic background of the *vgln-1* mutant was critical to expose the roles of many miRNAs and families of miRNAs in regulating the first larval stage of *C. elegans* development.

Although we were not able to determine a high confidence VGLN-1 binding motif using computational methods [RBPMotif (Kazan *et al.* 2010)], the enrichment of specific miRNA target sites among VGLN-1 target mRNAs could suggest that alternative methods of functional selectivity may exist. This enrichment and genetic interactions between *vgln-1* and miRNA/miRISC genes suggest potential collaboration between RNA binding protein VGLN-1 and miRNAs in regulating gene expressions for developmental functions, but there could be multiple potential models for such an interaction. In one model, this RNA-binding protein could potentially act as a corepressor of miRISCs to regulate the same mRNA targets. It is interesting to note that mammalian GW182 proteins, critical factors involved in miRISC-mediated gene silencing, actually contain RNA binding domains, albeit the roles of such domains have not yet been clearly demonstrated (Braun *et al.* 2013). Interestingly, the *C. elegans* AIN-1 (first GW182 protein determined to play an essential role in miRISC) and AIN-2 proteins lack such an RNA binding region (Ding *et al.* 2005; Rehwinkel *et al.* 2005; Zhang *et al.* 2007). Physical association and genetic interaction between RNA binding proteins and miRISCs have been detected in the worm (Zhang *et al.* 2007; Akay *et al.* 2013). However, physical interaction between VGLN-1 with miRISCs was not observed in our search for miRISC binding proteins (Zhang *et al.* 2007), nor in the search for VGLN-1 binding proteins, making the corepressor model unlikely. Lack of interaction between VGLN-1 and DCR-1 may also make a role of VGLN-1 in miRNA biogenesis unlikely (Duchaine *et al.* 2006). In addition, the genetic redundancy exhibited in *vgln-1*(*lf*); *miR-x*(*lf*) double mutants argues that the two systems act in parallel (nonindependent) mechanisms. Alternatively, and more consistent with the genetic data, VGLN-1 and miRNA may act in parallel to regulate the expression of many genes, some of which could be common targets, for a specific physiological function. The genetic data does not implicate that the regulations need to be at the same level even for the shared targets, particularly since both miRNAs and VGLN-1 are known to regulate gene expression at levels other than mRNA stability. Specifically, a recent paper indicated that Vigilin regulates gene expression mainly through inhibiting translation (Mobin *et al.* 2016). mRNA localization has also been suggested to be involved in VGLN-1 function (Gelin-Licht *et al.* 2012). These regulations might be the key reason that we were not successful in determining the functional relationship between VGLN-1 and miRNAs by analyzing the mRNA levels of selective genes. Growth and genetic background differences between mutant strains may have also presented an obstacle to obtain consistent data at specific developmental stages. Therefore, future mechanistic studies that analyze protein level changes for specific mRNA targets may be required to elucidate the potential regulatory roles of VGLN-1 and miRNAs on these targets at specific developmental stages.

## Supplementary Material

Supplemental material is available online at www.g3journal.org/lookup/suppl/doi:10.1534/g3.117.043414/-/DC1.

## References

[bib1] Afgan E. Baker D. van den Beek M. Blankenberg D. Bouvier D., 2016 The Galaxy platform for accessible, reproducible and collaborative biomedical analyses: 2016 update. Nucleic Acids Res. 44: W3–W10. 27137889 10.1093/nar/gkw343 PMC4987906

[bib2] Akay A. Craig A. Lehrbach N. Larance M. Pourkarimi E., 2013 RNA-binding protein GLD-1/quaking genetically interacts with the mir-35 and the let-7 miRNA pathways in *Caenorhabditis elegans*. Open Biol. 3: 130151. 24258276 10.1098/rsob.130151 PMC3843822

[bib3] Alvarez-Saavedra E. Horvitz H. R., 2010 Many families of *C. elegans* microRNAs are not essential for development or viability. Curr. Biol. 20: 367–373. 20096582 10.1016/j.cub.2009.12.051 PMC2844791

[bib4] Ambros V., 2004 The functions of animal microRNAs. Nature 431: 350–355. 15372042 10.1038/nature02871

[bib5] Anders S. Pyl P. T. Huber W., 2015 HTSeq–a Python framework to work with high-throughput sequencing data. Bioinformatics 31: 166–169. 25260700 10.1093/bioinformatics/btu638 PMC4287950

[bib6] Andrews S., 2016 FastQC—a quality control tool for high throughput sequence data. Babraham Bioinformatics. Available at: https://www.bioin-8formatics.babraham.ac.uk/projects/fastqc/.

[bib7] Bartel D. P., 2009 MicroRNAs: target recognition and regulatory functions. Cell 136: 215–233. 19167326 10.1016/j.cell.2009.01.002 PMC3794896

[bib8] Baugh L. R. Sternberg P. W., 2006 DAF-16/FOXO regulates transcription of cki-1/Cip/Kip and repression of lin-4 during *C. elegans* L1 Arrest. Curr. Biol. 16: 780–785. 16631585 10.1016/j.cub.2006.03.021

[bib9] Braun J. E. Huntzinger E. Izaurralde E., 2013 The role of GW182 proteins in miRNA-mediated gene silencing. Adv. Exp. Med. Biol. 768: 147–163. 23224969 10.1007/978-1-4614-5107-5_9

[bib10] Brenner S., 1974 The genetics of *Caenorhabditis elegans*. Genetics 77: 71–94. 4366476 10.1093/genetics/77.1.71 PMC1213120

[bib11] Brenner J. L. Jasiewicz K. L. Fahley A. F. Kemp B. J. Abbott A. L., 2010 Loss of individual microRNAs causes mutant phenotypes in sensitized genetic backgrounds in *C. elegans*. Curr. Biol. 20: 1321–1325. 20579881 10.1016/j.cub.2010.05.062 PMC2946380

[bib12] Brenner J. L. Kemp B. J. Abbott A. L., 2012 The mir-51 family of microRNAs functions in diverse regulatory pathways in *Caenorhabditis elegans*. PLoS One 7: e37185. 22615936 10.1371/journal.pone.0037185 PMC3353893

[bib13] Broughton J. P. Pasquinelli A. E., 2013 Identifying argonaute binding sites in *Caenorhabditis elegans* using iCLIP. Methods 63: 119–125. 23583680 10.1016/j.ymeth.2013.03.033 PMC3749250

[bib14] Cantacessi C. Loukas A. Campbell B. E. Mulvenna J. Ong E. K., 2009 Exploring transcriptional conservation between *Ancylostoma caninum* and *Haemonchus contortus* by oligonucleotide microarray and bioinformatic analyses. Mol. Cell. Probes 23: 1–9. 18977290 10.1016/j.mcp.2008.09.004

[bib15] Ceron J. Rual J.-F. Chandra A. Dupuy D. Vidal M., 2007 Large-scale RNAi screens identify novel genes that interact with the *C. elegans* retinoblastoma pathway as well as splicing-related components with synMuv B activity. BMC Dev. Biol. 7: 30. 17417969 10.1186/1471-213X-7-30 PMC1863419

[bib16] Ciafrè S. A. Galardi S., 2013 microRNAs and RNA-binding proteins. RNA Biol. 10: 934–942. 10.4161/rna.24641 PMC4111733 23696003

[bib17] Dickinson D. J. Ward J. D. Reiner D. J. Goldstein B., 2013 Engineering the *Caenorhabditis elegans* genome using Cas9-triggered homologous recombination. Nat. Methods 10: 1028–1034. 23995389 10.1038/nmeth.2641 PMC3905680

[bib18] Didiano D. Hobert O., 2006 Perfect seed pairing is not a generally reliable predictor for miRNA-target interactions. Nat. Struct. Mol. Biol. 13: 849–851. 16921378 10.1038/nsmb1138

[bib19] Didiano D. Hobert O., 2008 Molecular architecture of a miRNA-regulated 3′ UTR. RNA 14: 1297–1317. 18463285 10.1261/rna.1082708 PMC2441980

[bib20] Ding L. Spencer A. Morita K. Han M., 2005 The developmental timing regulator AIN-1 interacts with miRISCs and may target the argonaute protein ALG-1 to cytoplasmic P bodies in *C. elegans*. Mol. Cell 19: 437–447. 16109369 10.1016/j.molcel.2005.07.013

[bib21] Duchaine T. F. Wohlschlegel J. A. Kennedy S. Bei Y. Conte D. Jr., 2006 Functional proteomics reveals the biochemical niche of *C. elegans* DCR-1 in multiple small-RNA-mediated pathways. Cell 124: 343–354. 16439208 10.1016/j.cell.2005.11.036

[bib23] Frey S. Pool M. Seedorf M., 2001 Scp160p, an RNA-binding, polysome-associated protein, localizes to the endoplasmic reticulum of *Saccharomyces cerevisiae* in a microtubule-dependent manner. J. Biol. Chem. 276: 15905–15912. 11278502 10.1074/jbc.M009430200

[bib24] Frøkjær-Jensen C. Davis M. W. Ailion M. Jorgensen E. M., 2012 Improved Mos1-mediated transgenesis in *C. elegans*. Nat. Methods 9: 117–118. 22290181 10.1038/nmeth.1865 PMC3725292

[bib25] Garcia D. M. Baek D. Shin C. Bell G. W. Grimson A., 2011 Weak seed-pairing stability and high target-site abundance decrease the proficiency of lsy-6 and other microRNAs. Nat. Struct. Mol. Biol. 18: 1139–1146. 21909094 10.1038/nsmb.2115 PMC3190056

[bib26] Gelin-Licht R. Paliwal S. Conlon P. Levchenko A. Gerst J. E., 2012 Scp160-dependent mRNA trafficking mediates pheromone gradient sensing and chemotropism in yeast. Cell Reports 1: 483–494. 22832273 10.1016/j.celrep.2012.03.004 PMC3406329

[bib27] Graham L. Oram J., 1987 Identification and characterisation of a high density lipoprotein binding protein in cell membranes by ligand blotting. J. Biol. Chem. 262: 7439–7442. 3034894

[bib28] Grimson A. Srivastava M. Fahey B. Woodcroft B. J. Chiang H. R., 2008 Early origins and evolution of microRNAs and Piwi-interacting RNAs in animals. Nature 455: 1193–1197. 18830242 10.1038/nature07415 PMC3837422

[bib29] Hammell C. M. Karp X. Ambros V., 2009a A feedback circuit involving let-7-family miRNAs and DAF-12 integrates environmental signals and developmental timing in *Caenorhabditis elegans*. Proc Natl Acad Sci USA 106: 18668–18673. 19828440 10.1073/pnas.0908131106 PMC2774035

[bib30] Hammell C. M. Lubin I. Boag P. R. Blackwell T. K. Ambros V., 2009b nhl-2 modulates microRNA activity in *Caenorhabditis elegans*. Cell 136: 926–938. 19269369 10.1016/j.cell.2009.01.053 PMC2670343

[bib31] Hayes G. D. Ruvkun G., 2006 Misexpression of the *Caenorhabditis elegans* miRNA let-7 is sufficient to drive developmental programs. Cold Spring Harb. Symp. Quant. Biol. 71: 21–27. 17381276 10.1101/sqb.2006.71.018

[bib32] Hogan D. J. Riordan D. P. Gerber A. P. Herschlag D. Brown P. O., 2008 Diverse RNA-binding proteins interact with functionally related sets of RNAs, suggesting an extensive regulatory system. PLoS Biol. 6: e255. 18959479 10.1371/journal.pbio.0060255 PMC2573929

[bib33] Jan C. H. Friedman R. C. Ruby J. G. Bartel D. P., 2011 Formation, regulation and evolution of *Caenorhabditis elegans* 3′UTRs. Nature 469: 97–101. 21085120 10.1038/nature09616 PMC3057491

[bib34] Johnston R. J. Hobert O., 2003 A microRNA controlling left/right neuronal asymmetry in *Caenorhabditis elegans*. Nature 426: 845–849. 14685240 10.1038/nature02255

[bib35] Kamath R. S. Fraser A. G. Dong Y. Poulin G. Durbin R., 2003 Systematic functional analysis of the *Caenorhabditis elegans* genome using RNAi. Nature 421: 231–237. 12529635 10.1038/nature01278

[bib36] Kazan H. Ray D. Chan E. T. Hughes T. R. Morris Q., 2010 RNAcontext: a new method for learning the sequence and structure binding preferences of RNA-binding proteins. PLOS Comput. Biol. 6: e1000832. 20617199 10.1371/journal.pcbi.1000832 PMC2895634

[bib37] Knight C. G. Patel M. N. Azevedo R. B. R. Leroi A. M., 2002 A novel mode of ecdysozoan growth in *Caenorhabditis elegans*. Evol. Dev. 4: 16–27. 11871396 10.1046/j.1525-142x.2002.01058.x

[bib38] Lau N. C. Lim L. P. Weinstein E. G. Bartel D. P., 2001 An abundant class of tiny RNAs with probable regulatory roles in *Caenorhabditis elegans*. Science 294: 858–862. 11679671 10.1126/science.1065062

[bib39] Lee R. C. Ambros V., 2001 An extensive class of small RNAs in *Caenorhabditis elegans*. Science 294: 862–864. 11679672 10.1126/science.1065329

[bib40] Li A.-M. Watson A. Fridovich-Keil J. L., 2003 Scp160p associates with specific mRNAs in yeast. Nucleic Acids Res. 31: 1830–1837. 12654998 10.1093/nar/gkg284 PMC152800

[bib41] Li Y. Kowdley K. V., 2012 MicroRNAs in common human diseases. Genomics Proteomics Bioinformatics 10: 246–253. 23200134 10.1016/j.gpb.2012.07.005 PMC3611977

[bib42] Love M. I. Huber W. Anders S., 2014 Moderated estimation of fold change and dispersion for RNA-seq data with DESeq2. Genome Biol. 15: 550. 25516281 10.1186/s13059-014-0550-8 PMC4302049

[bib43] Martinez N. J. Ow M. C. Reece-hoyes J. S. Barrasa M. I. Ambros V. R., 2008 Genome-scale spatiotemporal analysis of *Caenorhabditis elegans* microRNA promoter activity. 18: 2005–2015. 10.1101/gr.083055.108 PMC2593583 18981266

[bib44] Massirer K. B. Perez S. G. Mondol V. Pasquinelli A. E., 2012 The miR-35–41 family of microRNAs regulates RNAi sensitivity in *Caenorhabditis elegans*. PLoS Genet. 8: e1002536. 22412382 10.1371/journal.pgen.1002536 PMC3297572

[bib45] McGhee J. D. Sleumer M. C. Bilenky M. Wong K. McKay S. J., 2007 The ELT-2 GATA-factor and the global regulation of transcription in the *C. elegans* intestine. Dev. Biol. 302: 627–645. 17113066 10.1016/j.ydbio.2006.10.024

[bib46] Mendell J. T. Olson E. N., 2012 MicroRNAs in stress signaling and human disease. Cell 148: 1172–1187. 22424228 10.1016/j.cell.2012.02.005 PMC3308137

[bib47] Merritt C., 2010 Transgenic solutions for the germline. (February 8, 2010), *WormBook* , ed. The *C. elegans* Research Community, WormBook, /10.1895/wormbook.1.148.1, http://www.wormbook.org. 10.1895/wormbook.1.148.1 PMC4966531 20169625

[bib48] Miska E. A. Alvarez-Saavedra E. Abbott A. L. Lau N. C. Hellman A. B., 2007 Most *Caenorhabditis elegans* microRNAs are individually not essential for development or viability. PLoS Genet. 3: e215. 18085825 10.1371/journal.pgen.0030215 PMC2134938

[bib49] Mitchell S. F. Jain S. She M. Parker R., 2013 Global analysis of yeast mRNPs. Nat. Struct. Mol. Biol. 20: 127–133. 23222640 10.1038/nsmb.2468 PMC3537908

[bib50] Mobin M. B. Gerstberger S. Teupser D. Campana B. Charisse K., 2016 The RNA-binding protein vigilin regulates VLDL secretion through modulation of Apob mRNA translation. Nat. Commun. 7: 12848. 27665711 10.1038/ncomms12848 PMC5052685

[bib51] Molyneux S. D. Waterhouse P. D. Shelton D. Shao Y. W. Watling C. M., 2014 Human somatic cell mutagenesis creates genetically tractable sarcomas. Nat. Genet. 46: 964–972. 25129143 10.1038/ng.3065

[bib52] Moore M. J. Zhang C. Gantman E. C. Mele A. Darnell J. C., 2014 Mapping argonaute and conventional RNA-binding protein interactions with RNA at single-nucleotide resolution using HITS-CLIP and CIMS analysis. Nat. Protoc. 9: 263–293. 24407355 10.1038/nprot.2014.012 PMC4156013

[bib53] Orellana E. Kasinski A., 2015 MicroRNAs in cancer: a historical perspective on the path from discovery to therapy. Cancers (Basel) 7: 1388–1405. 26226002 10.3390/cancers7030842 PMC4586775

[bib54] Otsuka H. Gotoh Y. Komeno T. Ono T. Kawasaki Y., 2014 Aralin, a type II ribosome-inactivating protein from *Aralia elata*, exhibits selective anticancer activity through the processed form of a 110-kDa high-density lipoprotein-binding protein: a promising anticancer drug. Biochem. Biophys. Res. Commun. 453: 117–123. 25261720 10.1016/j.bbrc.2014.09.067

[bib55] Reboul J. Vaglio P. Rual J.-F. Lamesch P. Martinez M., 2003 *C. elegans* ORFeome version 1.1: experimental verification of the genome annotation and resource for proteome-scale protein expression. Nat. Genet. 34: 35–41. 12679813 10.1038/ng1140

[bib56] Rehwinkel J. Behm-Ansmant I. Gatfield D. Izaurralde E., 2005 A crucial role for GW182 and the DCP1:DCP2 decapping complex in miRNA-mediated gene silencing. RNA 11: 1640–1647. 16177138 10.1261/rna.2191905 PMC1370850

[bib57] Ren Z. Veksler-Lublinsky I. Morrissey D. Ambros V., 2016 Staufen negatively modulates microRNA activity in *Caenorhabditis elegans*. G3 Bethesda 6: 1227–1237. 26921297 10.1534/g3.116.027300 PMC4856075

[bib58] Rohwedel J. Kügler S. Engebrecht T. Purschke W. Müller P. K., 2003 Evidence for posttranscriptional regulation of the multi K homology domain protein vigilin by a small peptide encoded in the 5′ leader sequence. Cell. Mol. life Sci. 60: 1705–1715. 14504658 10.1007/s00018-003-3134-4 PMC11138898

[bib59] Shaw W. R. Armisen J. Lehrbach N. J. Miska E. A., 2010 The conserved miR-51 microRNA family is redundantly required for embryonic development and pharynx attachment in *Caenorhabditis elegans*. Genetics 185: 897–905. 20421599 10.1534/genetics.110.117515 PMC2900971

[bib60] Simmer F. Moorman C. van der Linden A. M. Kuijk E. van den Berghe P. V. E., 2003 Genome-wide RNAi of *C. elegans* using the hypersensitive rrf-3 strain reveals novel gene functions. PLoS Biol. 1: E12. 14551910 10.1371/journal.pbio.0000012 PMC212692

[bib61] Spencer W. C. Zeller G. Watson J. D. Henz S. R. Watkins K. L., 2011 A spatial and temporal map of *C. elegans* gene expression. Genome Res. 21: 325–341. 21177967 10.1101/gr.114595.110 PMC3032935

[bib62] Stiernagle T., 2006 Maintenance of *C. elegans*. (February 11, 2006), *WormBook*, ed. The *C. elegans* Research Community, WormBook, /10.1895/wormbook.1.101.1, http://www.wormbook.org. 10.1895/wormbook.1.101.1 PMC4781397 18050451

[bib63] Trapnell C. Pachter L. Salzberg S. L., 2009 TopHat: discovering splice junctions with RNA-Seq. Bioinformatics 25: 1105–1111. 19289445 10.1093/bioinformatics/btp120 PMC2672628

[bib64] Trapnell C. Hendrickson D. G. Sauvageau M. Goff L. Rinn J. L., 2013 Differential analysis of gene regulation at transcript resolution with RNA-seq. Nat. Biotechnol. 31: 46–53. 23222703 10.1038/nbt.2450 PMC3869392

[bib65] van Kouwenhove M. Kedde M. Agami R., 2011 MicroRNA regulation by RNA-binding proteins and its implications for cancer. Nat. Rev. Cancer 11: 644–656. 21822212 10.1038/nrc3107

[bib66] Vella M. C. Slack F. J., 2005 *C. elegans* microRNAs. (September 21, 2005), WormBook, ed. The C. elegans Research Community, WormBook, /10.1895/wormbook.1.26.1, http://www.wormbook.org. 10.1895/wormbook.1.26.1 PMC4781108 18050425

[bib67] Vidigal J. A. Ventura A., 2014 The biological functions of miRNAs: lessons from in vivo studies. Trends Cell Biol. 25: 137–147. 25484347 10.1016/j.tcb.2014.11.004 PMC4344861

[bib68] Wang Q. Zhang Z. Blackwell K. Carmichael G. G., 2005 Vigilins bind to promiscuously A-to-I-Edited RNAs and are involved in the formation of heterochromatin. Curr Biol. 15:384–391. 15723802 10.1016/j.cub.2005.01.046

[bib69] Weaver B. P. Zabinsky R. Weaver Y. M. Lee E. S. Xue D., 2014 CED-3 caspase acts with miRNAs to regulate non-apoptotic gene expression dynamics for robust development in *C. elegans*. eLife 3: e04265. 25432023 10.7554/eLife.04265 PMC4279084

[bib70] Weber V. Wernitznig A. Hager G. Harata M. Frank P., 1997 Purification and nucleic-acid-binding properties of a *Saccharomyces cerevisiae* protein involved in the control of ploidy. Eur. J. Biochem. 249: 309–317. 9363784 10.1111/j.1432-1033.1997.00309.x

[bib71] Woo H.-H. Yi X. Lamb T. Menzl I. Baker T., 2011 Posttranscriptional suppression of proto-oncogene c-fms expression by Vigilin in breast cancer. Mol. Cell. Biol. 31: 215–225. 20974809 10.1128/MCB.01031-10 PMC3019847

[bib72] Yang W. L. Wei L. Huang W. Q. Li R. Shen W. Y., 2014 Vigilin is overexpressed in hepatocellular carcinoma and is required for HCC cell proliferation and tumor growth. Oncol. Rep. 31: 2328–2334. 24676454 10.3892/or.2014.3111

[bib73] Zhang H. Kim A. Abraham N. Khan L. A. Hall D. H., 2012 Clathrin and AP-1 regulate apical polarity and lumen formation during *C. elegans* tubulogenesis. Development 139: 2071–2083. 22535410 10.1242/dev.077347 PMC3347694

[bib74] Zhang L. Ding L. Cheung T. H. Dong M. Q. Chen J., 2007 Systematic identification of *C. elegans* miRISC proteins, miRNAs, and mRNA targets by their interactions with GW182 proteins AIN-1 and AIN-2. Mol. Cell 28: 598–613. 18042455 10.1016/j.molcel.2007.09.014 PMC2186060

[bib75] Zhao J. Ohsumi T. K. Kung J. T. Ogawa Y. Grau D. J., 2010 Genome-wide identification of polycomb-associated RNAs by RIP-seq. Mol. Cell 40: 939–953. 21172659 10.1016/j.molcel.2010.12.011 PMC3021903

[bib76] Zhou J. Wang Q. Chen L.-L. Carmichael G. G., 2008 On the mechanism of induction of heterochromatin by the RNA-binding protein vigilin. RNA 14: 1773–1781. 18648073 10.1261/rna.1036308 PMC2525967

[bib77] Zhu H. Sewell A. K. Han M., 2015 Intestinal apical polarity mediates regulation of TORC1 by glucosylceramide in *C. elegans*. Genes Dev. 29: 1218–1223. 26109047 10.1101/gad.263483.115 PMC4495394

