# Supplementary material for: RNA Binding Protein Vigilin Collaborates with miRNAs To Regulate Gene Expression for Caenorhabditis elegans Larval Development
Source: G3 (Bethesda). 2017 Jun 2;7(8):2511–8. doi: 10.1534/g3.117.043414 (PMC5555458; doi:10.1534/g3.117.043414)
Supplement: Supplementary file 1 [file 2511FileS1.pdf]

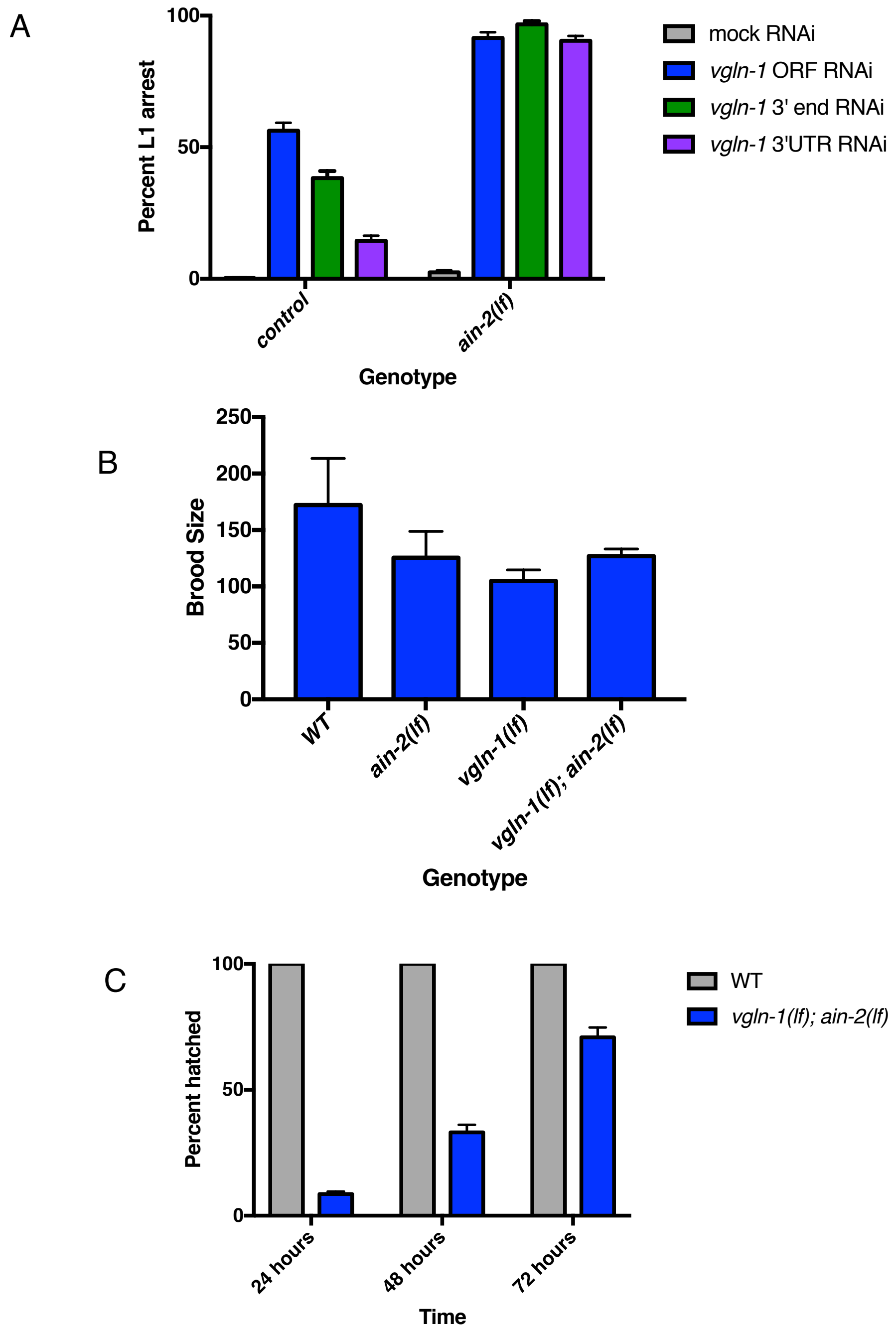

Supplemental Figure 1. Vigilin is essential for development and reproduction when the miRISC is compromised. A. Percentage of hatched animals arrested at the early L1 stage of indicated genotype or treatment. Multiple RNAi constructs targeted against the *vgln-1* gene replicate the synthetic L1 arrest. Different constructs are color-coded and error bars represent standard error of proportion (SEM). N > 160 worms counted under each condition. B. Average brood size is not dramatically reduced in *vgln-1* or *vgln-1;ain-2*. Error bars represent SEM. C. Hatching rate is severely reduced in *vgln-1;ain-2* double mutant. Error bars represent SEM.

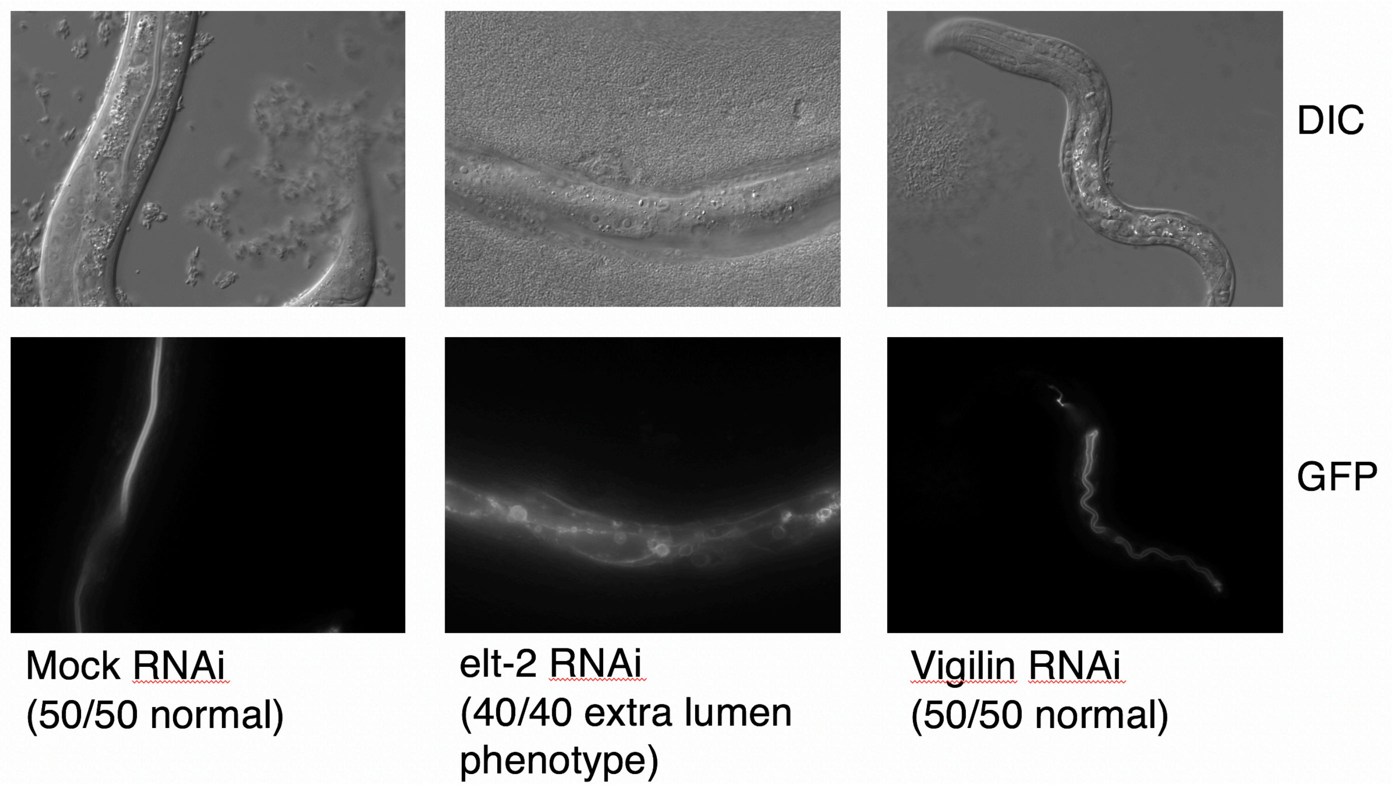

Supplemental Figure 2. Intestinal tubulogenesis is not disrupted by *vgln-1* RNAi. Unlike *elt-2* RNAi, that caused defects in intestinal tubulogenesis (indicated by ERM-1::GFP). *vgln-1* RNAi did not disrupt intestinal tubulogenesis.

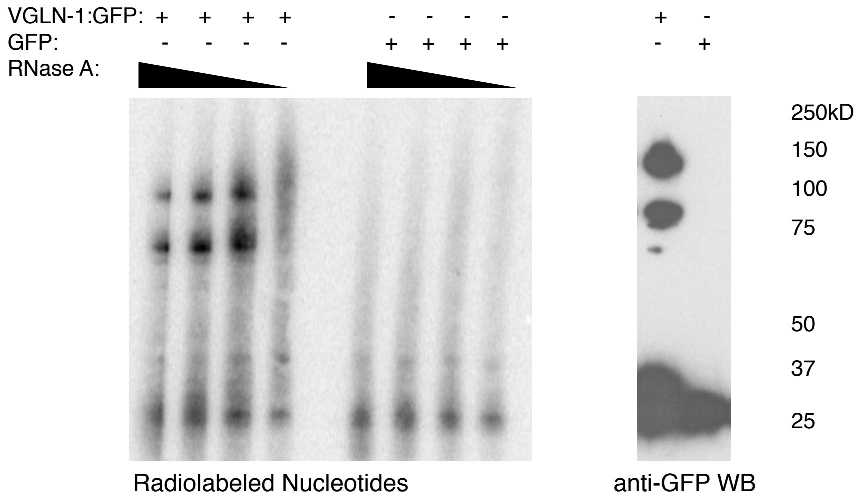

Supplemental Figure 3. Cross linking and immunoprecipitation reveals the protein RNA complex is sensitive to increasing concentrations of RNase.

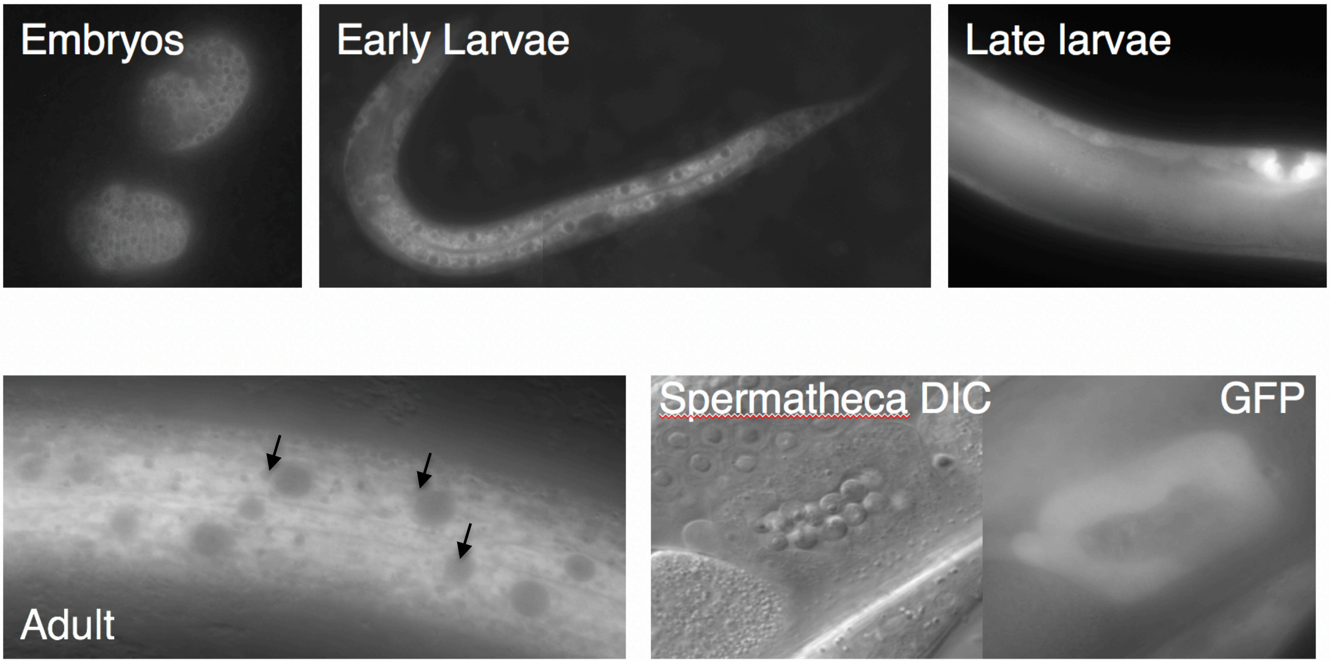

Supplemental Figure 4. VGLN-1::GFP is expressed broadly throughout development. Representative images show the embryo, early larvae (to highlight expression in the head and intestine), late larvae (to highlight expression in the vulva), and adult (to highlight expression in muscle, hypodermis, and somatic spermatheca). In the intestine, muscle, and hypodermis VGLN-1::GFP appears to be excluded from nuclei (black arrows highlight three examples).
